# Curative Effect and Mechanism of Guiren Runchang Granules on Morphine-Induced Slow Transit Constipation in Mice

**DOI:** 10.1155/2020/5493192

**Published:** 2020-09-23

**Authors:** Yihan Sun, Chengqiu Yan, Shifeng Jin, Chong Shi, Jingming Zhao, Guofeng Li

**Affiliations:** ^1^School of Traditional Chinese Medicine, Changchun University of Chinese Medicine, Changchun, Jilin 130117, China; ^2^Anorectal Department, Affiliated Hospital of Changchun University of TCM, Changchun, Jilin 130021, China

## Abstract

Recent studies have identified the curative effects of traditional Chinese medicine for constipation. The mechanism of action of Guiren Runchang granules (GRGs) in the treatment of slow transit constipation (STC) was evaluated in this study. Here, we assessed the efficacy of GRG by comparing the differences in fecal characteristics, stool weight, and intestinal transit rate (ITR) among 6 groups (*n* = 12/group), which were administered three concentrations of GRG, mosapride, and saline. The influence of GRG on the SCF/c-kit pathway, AQP4, and serum motilin of mice was assessed through ELISA, western blot, and immunohistochemical analysis. The dry weight of mouse feces at 24 hr and ITR in the MD (medium-dose GRG; 9.44 g/kg/d) and HD (high-dose GRG; 18.88 g/kg/d) groups was higher than that in the MC (model control) group. The serum motilin of morphine-induced mice level was lower in the MC group than in the NC (normal control) group, and this condition was improved in the HD group. The HD group expressed significantly higher levels of SCF and c-kit protein but lower levels of AQP4 and simultaneously presented more SCF-positive and c-kit-positive cells. However, no differences in the serum SCF level were found among the six groups. Certain concentrations of GRG are effective in STC mice, the potential mechanism of which may be associated with repairing the SCF/c-kit pathway and reducing the expression of AQP4 in the colon. GRG improved the serum motilin level but had no influence on the serum SCF level.

## 1. Introduction

Slow transit constipation (STC), often characterized by infrequent bowel movements and hard stools, is the most common type of chronic constipation. Related studies show that the global incidence of chronic constipation is increasing yearly [1–3]. Chronic constipation is frequently associated with pain, inconvenience, and concurrent diseases, especially mental disorders [4]. Although a number of medical treatment methods are available, poor curative outcomes and adverse effects are still widespread. Surgical therapy, as a last resort after the failure of other therapies, is not widely accepted due to safety concerns and secondary injury [5]. Hence, seeking alternative therapies for STC is of great significance for patients.

Various factors can lead to STC, including food type, genetic factors, depression, medications, and other colorectal diseases [6]. To date, there is no consensus on the pathogenesis of STC. Changes in gastrointestinal peptide concentration might be one potential cause. Previous studies have shown that changes in serum motilin levels are highly associated with the occurrence of STC [7–9]. Somatostatin, another kind of gastrointestinal peptide, can reduce intestinal motility and secretory activity [10]. An array of previous studies demonstrated that a decline in the number of interstitial cells of Cajal (ICCs) may lead to STC [11, 12]. c-kit, combined with its ligand stem cell factor (SCF), is responsible for ICC quantity, and blocking the SCF/c-kit signal pathway could indirectly cause STC [13, 14]. In addition, the level of aquaporin, which is expressed and scattered throughout colon tissue, may play an important role in the development of STC. Thus, demonstrating the mechanisms of different pathways is necessary for verifying the efficacy of a therapeutic method against this disease.

As an alternative therapy for constipation, traditional Chinese medicine (TCM) is safe, universally applied, and effective [15]. Guiren Runchang granule (GRG), a Chinese herbal compound, has been applied to patients with STC at the Traditional Chinese Medicine Hospital of Jilin Province (Jilin China) for decades. The major ingredients of GRG include angelica, *Cistanche*, *Magnolia*, and Fructus Aurantii. However, the precise mechanism by which GRG improves STC remains unclear. In this study, we investigated the curative effect of GRG on STC mice and revealed its mechanism by measuring the serum motilin concentration and the expression of c-kit, SCF, and aquaporin in the colon.

## 2. Materials and Methods

### 2.1. Animals

Seventy-two healthy SPF Kunming (KM) mice (generated from Swiss mice, 6 weeks of age, half male and female), weighing 19 ± 1 g, were purchased from Liaoning Chang Sheng Biotechnology Co. (Liaoning, China) and housed at the Laboratory Animal Center of Changchun University of Traditional Chinese Medicine (Jilin, China). All animals were maintained in one laboratory at 20∼25°C and 40%∼60% relative humidity under a 12 h light/dark cycle. All mice were provided ordinary feed and water *ad libitum*. All the experiments adhered to the Guide for Care and Use of Laboratory Animals of Changchun University of Traditional Chinese Medicine (Changchun, China).

### 2.2. Drugs

The Traditional Chinese Medicine Hospital of Jilin Province (Jilin China) provided all the herbs for this study. GRG was prepared as follows: 10 g angelica, 25 g raw atractylodes, 15 g peach kernel, 15 g *Cistanche*, 25 g Fructus Aurantii, 10 g magnolia, 15 g pollen, *Typhae*, 12 g trogopterus dung, 20 g *Trichosanthes kirilowii* Maxim, and 6 g *Liquorice* dry powder were mixed and boiled in 2000 ml deionized (DI) water until the concentration of the herbal mixture attained 4 g/ml. The extract was processed into granules by the TCM pharmacy. Mosapride citrate tablets were purchased from Yabao Pharmaceutical Group Co. Ltd. (H20090158; Shanxi, China), and morphine hydrochloride injection was provided by Changchun University of Traditional Chinese Medicine (Changchun, China) after related approval.

### 2.3. UPLC Analysis of GRG

**Figure 1:**
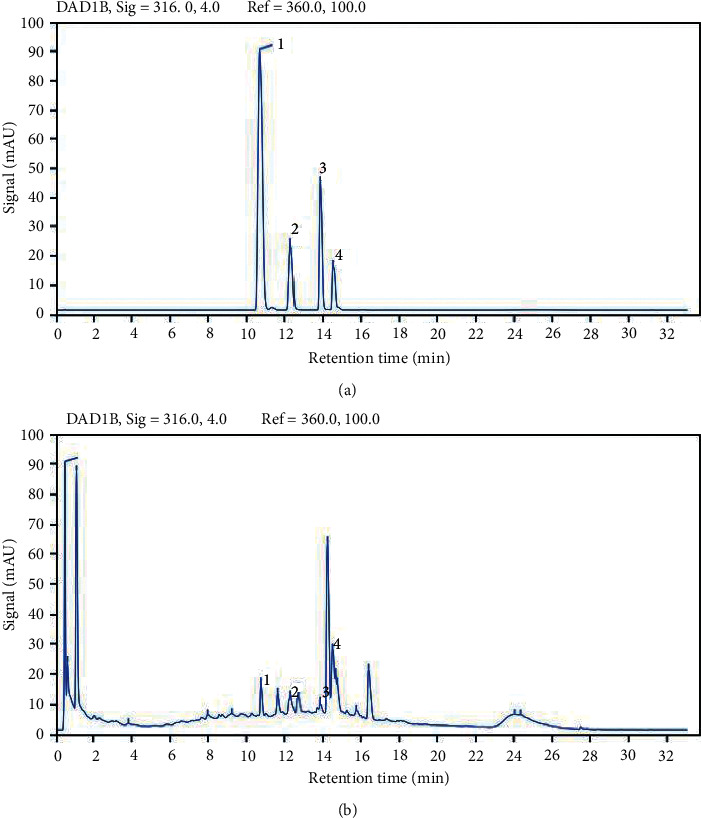
UPLC chromatogram of Guiren Runchang granules (GRG). (a) UPLC chromatogram of mixed reference substance. (b) UPLC chromatogram of GRG extracts.

Ultra performance liquid chromatography (UPLC) analysis was applied to evaluate the quality and content of the main components extracted from GRG. The assay was performed by Agilent 1290 Infinity II UPLC-DAD (Agilent Technologies, CA, USA). Water with 0.1% glacial acetic acid (A), combined with acetonitrile (B), were the main ingredients of the mobile phase system. Profile of the gradient elution was as follows: 0∼3 min, 2% B; 3∼5 min, 2%∼5% B; 5∼8 min, 5%∼10% B; 8∼13 min, 10%∼18% B; 13∼16 min, 18%∼19.7% B; 16∼20 min, 19.7%∼21% B; 20∼21 min, 21% B; 21∼33 min, 21%∼60% B. The UPLC separation was performed on Agilent ZORBAX SB-C18 column (100 × 2.1 mm, 2.6 *μ*m, thermo) and the mobile phase flow rate was set as 0.3 ml/min. During the whole process of detection, 30°C was a better column temperature to ensure the resolution and the spectrum was set at 316 nm. The reference substances were purchased from Chengdu GLP-Biotechnology Co., Ltd. (Chengdu, China), and listed as follows: ferulic acid (>98% purity, CAS:1135-24-6), liquiritin (>98% purity, CAS: 551-15-5), acteoside (>98% purity, CAS: 61276-17-3), naringin (>98% purity, CAS:10236-47-2). Four components were identified and UPLC chromatogram of GRG is shown in Figure 1.

### 2.4. Study Design

Thirty-six male mice and 36 female mice were randomly allocated to six groups (12 mice per group with 6F + 6M): a normal control (NC) group, a model control (MC) group, a positive control (PC) group, a low-dose GRG (LD) group, a medium-dose GRG (MD) group, and a high-dose GRG (HD) group. The STC mouse model was developed by administering continuous subcutaneous injections of morphine hydrochloride (2.5 mg/kg/d) for 45 days, as previously described [16]. After model establishment, the LD, MD, and HD groups were treated by gavage with 4.72 g/kg/d, 9.44 g/kg/d, and 18.88 g/kg/d GRG, respectively, comparable to the clinical doses applied in adults [17]. The PC group was administered with mosapride suspension (1.15 g/kg/d), and the NC and MC groups were treated with the same volume of saline. All treatments were performed once a day and lasted for 14 days. During this period, all mice had free access to food and water. The mice were weighed every other day, and the drugs dosage was adjusted according to weight.

### 2.5. Stool Weight and Intestinal Transit Rate

Twenty-four hours of fecal material was gathered once a week before and after the treatment and then dried and weighed. To measure the intestinal transit rate, all mice were given 0.5 ml of 5% activated carbon suspension by intragastric administration. Half an hour later, the mice were euthanized, and the whole intestinal tissue, from the pylorus to the anus, was dissected out and gently straightened without tension. The total length of the intestine, together with the dyed portion, was measured. The intestinal transit rate was calculated as the length of the stained part/length of the total intestine ×100%.

### 2.6. ELISA

A 0.3 ml blood sample was drawn from the posterior orbital venous plexus before the mice were sacrificed. After 2 hours of incubation at 4°C, the samples were centrifuged for 10 min (3000 rpm) to obtain the supernatant. The serum motilin level and the SCF level of the blood serum were measured with two mouse ELISA kits (R&D systems, Minneapolis, MN, USA).

### 2.7. Western Blot Analysis

Colon tissue samples from each group were cut into pieces and homogenized with lysis buffer by blending on ice for 20 min. The homogenates were centrifuged at 12000 rpm (4°C) for 10 min, and the liquid supernatant was subsequently extracted. A bicinchoninic acid (BCA) protein assay was used to measure the total protein concentration, after which all the samples were normalized and aliquoted (200 *μ*L per test tube). The samples were mixed with loading buffer, boiled, and centrifuged at 2000 rpm for 30 seconds. The liquid supernatant was collected and separated by SDS-PAGE. Proteins were transferred onto polyvinylidene difluoride membranes for 100 min (Millipore, Billerica, MA, USA). All the membranes were blocked with 5% skim milk powder solution (2 g skim milk powder, 40 ml TBST) overnight at 4°C and then incubated with primary SCF antibody (lot #I1917, Santa Cruz), AQP4 antibody (lot #D2318, Santa Cruz), and c-kit antibody (lot #C2919, Santa Cruz) at 37°C for 2 h. After washing with TBST, the membranes were incubated with secondary antibody (lot #K2818, Santa Cruz) at room temperature and visualized with a chemiluminescence kit (Millipore). The imaging results were analyzed by Image J software (National Institutes of Health, Bethesda, MD).

### 2.8. Immunohistochemical Analysis

Samples embedded in paraffin were processed into 4 *µ*m thick slices. The sections were incubated with primary SCF antibody (lot #I1917, Santa Cruz) and c-kit antibody (lot #C2919, Santa Cruz) overnight at 4°C after deparaffinization, hydration, antigen retrieval, and blockade. Secondary antibodies (lot #K2818, Santa Cruz) were added to the sections, which were then washed with PBS buffer solution and reacted for 1 h at room temperature. Chromogenic reaction was induced with diaminobenzidine for 10 min under a microscope, followed by counterstaining with hematoxylin. NIS-Elements BR software (Nikon Corporation, JPN) was used to analyze the images.

### 2.9. Statistical Analysis

SPSS 25.0 (IBM, Armonk, NY, USA) was used for data analysis. The experimental results are presented as the mean ± standard deviation. One-way analysis of variance combined with multiple comparisons was used to analyze differences between the samples. *P* < 0.05 was considered statistically significant.

## 3. Results and Discussion

### 3.1. Results

#### 3.1.1. UPLC Profile of GRG

UPLC chromatograms of reference substances and GRG were, respectively, exhibited in Figures 1(a) and 1(b). Through the UPLC assay, five known chemical monomers and the concentration of them in GRG dry powder were identified. Detailed information was listed as follows: (1) ferulic acid (113.836 *μ*g/g); (2) liquiritin (490.883 *μ*g/g); (3) acteoside (167.074 *μ*g/g); (4) naringin (3410.776 *μ*g/g).

#### 3.1.2. Mouse Model

**Figure 2:**
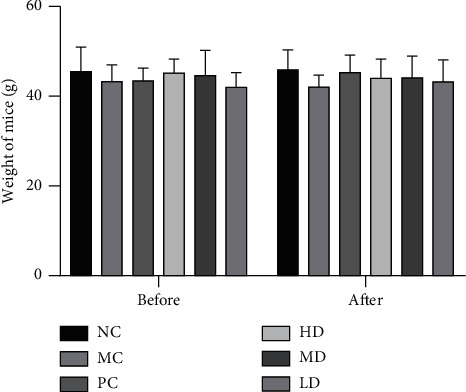
Weights of the mice before and after the treatment. The colors indicate different groups.

No mice died during the whole experiment. Compared with the NC group, the MC mice showed less lustrousness and smooth back hair and less active back hair. There was no significant difference in weight among all six groups before or after treatment (*P* > 0.05) (Figure 2). No significant difference was found between males and females (*P* > 0.05).

#### 3.1.3. Stool Changes

**Figure 3:**
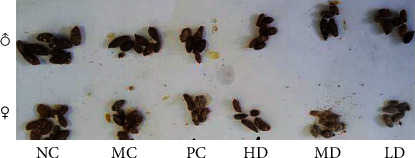
Stool characteristics (59th day).

**Figure 4:**
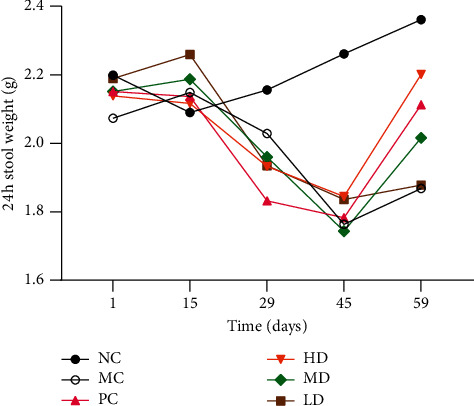
Time-dependent changes in stool weight. The colors indicate different groups.

The feces of the MC group were drier, lighter, and smaller than those of the NC group. There was no significant difference in fecal characteristics among the PC, LD, MD, and HD groups (Figure 3). The feces of the females were smaller and drier than that of males in all six groups. Before the procedure of model establishment, no differences existed among the groups (*P* > 0.05). Forty-five days after injection of morphine, the dry weight of feces at 24 h in the MC group was significantly lower than that in the NC group (1.76 ± 0.13 versus 2.26 ± 0.16, (*P* < 0.01)). After 14 days of gavage, the dry weight of feces of the MD group (2.20 ± 0.12 versus 1.87 ± 0.18, *P* < 0.05), combined with the HD and PC groups (2.20 ± 0.12 versus 1.87 ± 0.18; 2.11 ± 0.15 versus 1.87 ± 0.18, (*P* < 0.01)), was higher than that of the MC group, whereas the dry weight of feces of all the other groups was lower than that of the NC group (*P* < 0.05) (Figure 4).

#### 3.1.4. ITR Change

**Figure 5:**
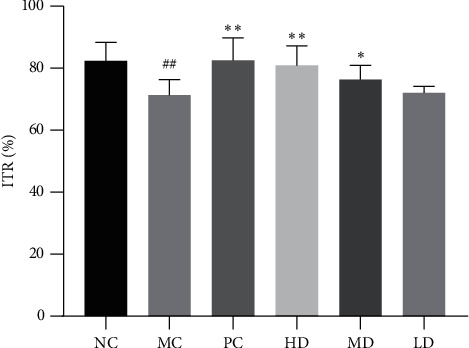
ITR of the mice after treatment (*n* = 12). ^##^*P* < 0.01 versus NC group; ^*∗*^*P* < 0.05 and ^*∗∗*^*P* < 0.01 versus MC group.

The ITR of the MC group was markedly lower than that of the NC group (*P* < 0.01). The ITR of the MD group was higher than that of the MC group (*P* < 0.05), and the ITR of the PC and HD groups was elevated in the meanwhile (*P* < 0.01). The difference in ITR between the HD group and the LD group was also significant (*P* < 0.01) (Figure 5).

#### 3.1.5. Effect of GRG on Motilin

**Figure 6:**
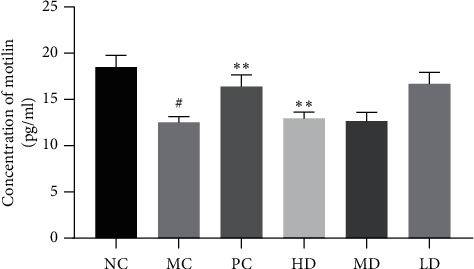
Differences of serum's motilin in mice (*n* = 12). One-way ANOVA and post hoc test demonstrated that no statistical difference exists between HD and PC's data (*P* > 0.05), also among MC, LD, and MD (*P* > 0.05). However, significant differences were presented between HD and MC (^#^*P* < 0.05 versus NC group), as well as between HD and MD's data (^*∗∗*^*P* < 0.01 versus MC group).

The serum motilin level of the NC group was higher than that of the MC group (*P* < 0.05). The serum motilin level was significantly higher in the PC and HD groups than in the MC group (*P* < 0.01), while there was no significant difference between the PC and HD groups. However, the serum motilin level was not elevated in either the MD group or the LD group (*P* < 0.05) (Figure 6).

#### 3.1.6. Influence on SCF

**Figure 7:**
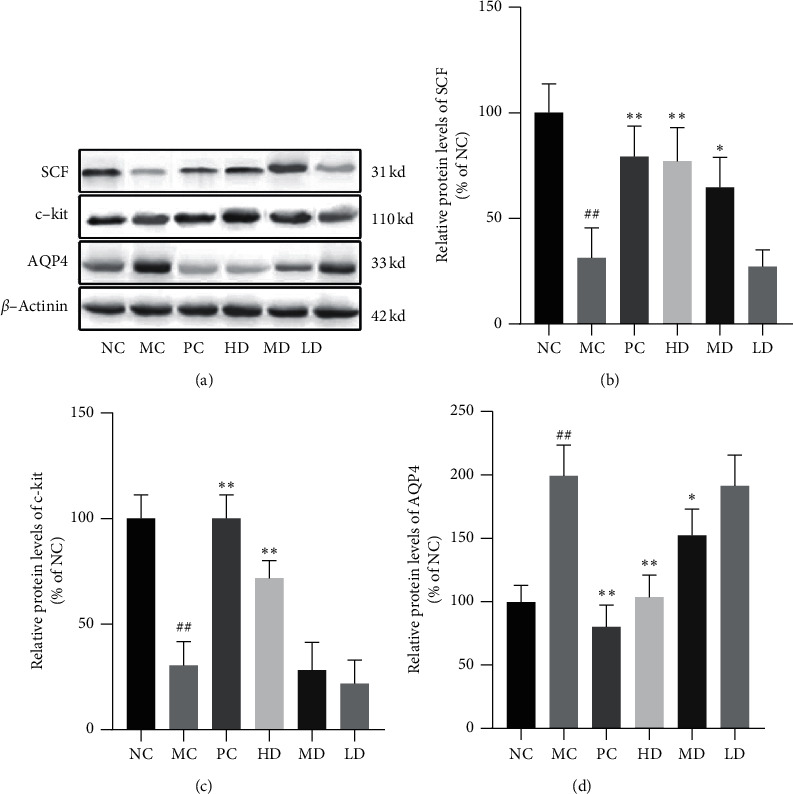
(a) Result of western blot analysis. (b) Relative protein levels of SCF (% of NC). The MC group presented an obvious lower specific ratio than the NC group (31.25 ± 14.23 versus 100.00 ± 13.61). In comparison with MC group, HD and PC group showed significantly higher ratio (77.09 ± 15.77 versus 31.25 ± 14.23; 79.17 ± 14.43 versus 31.25 ± 14.23). (c) Relative protein levels of c-kit (% of NC). MC group presented strikingly lower ratio than NC group (30.44 ± 11.23 versus 100.00 ± 11.22). In comparison to MC group, the ratio of PC and HD groups was markedly higher (30.44 ± 11.23 versus 100.00 ± 11.22; 30.44 ± 11.23 versus 71.74 ± 8.32), while MD and LD groups presented no difference (*P* > 0.05). (d) Relative protein levels of AQP4 (% of NC). The MC group had an obvious higher ratio than the NC group (199.22 ± 24.29 versus 99.61 ± 13.34). In contrast with the MC group, both PC and HD groups showed significant differences (199.22 ± 24.29 versus 80.08 ± 17.32; 199.22 ± 24.29 versus 103.52 ± 17.32). The MD group presented difference as well (199.22 ± 24.29 versus 152.35 ± 20.67). Quantification data was normalized to the corresponding *β*-actinin levels. Data are expressed as percentage of NC and mean ± SD (*n* = 4). ^##^*P* < 0.01 versus NC group; ^*∗*^*P* < 0.05 and ^*∗∗*^*P* < 0.01 versus MC group.

**Figure 8:**
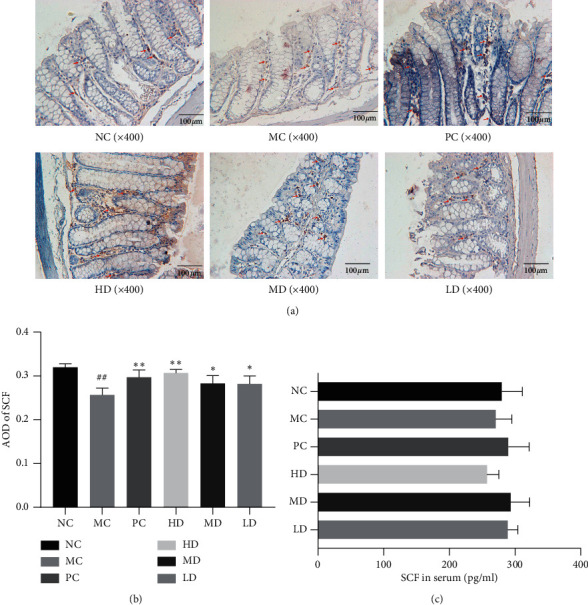
(a) Immunohistochemical analysis of SCF. (b) AOD of SCF. The NC group's colon SCF AOD value was markedly higher than MC group (0.320 ± 0.008 versus 0.257 ± 0.015). The AOD value were increased apparently in PC and HD group compared to MC group (0.297 ± 0.017 versus 12.51 ± 0.61; 0.307 ± 0.008 versus 12.51 ± 0.61). ^##^*P* < 0.01 versus NC group; ^*∗*^*P* < 0.05 and ^*∗∗*^*P* < 0.01 versus MC group. (c) Serum SCF assays (*n* = 12). No statistical difference was found between six groups (*P* > 0.05).

The results of western blot analysis indicated that the colons of NC group mice presented markedly higher SCF concentrations than the colons of the MC group mice (*P* < 0.01). Both the MD and HD groups expressed higher levels of SCF than the MC group (*P* < 0.01). No obvious difference was found between the HD and PC groups (*P* > 0.05) (Figures 7(a) and 7(b)). Similarly, immunohistochemical analysis showed that SCF-positive cells were scattered within the mesenchyme of colon glands. SCF-positive cells were less abundant and more dispersed in the MC group than in the NC group. More SCF-positive cells were found in the HD group than in the PC group. There was no significant difference between the PC and MD groups. The LD group showed a sparser distribution of SCF-positive cells but a more intensive distribution than the MC group (Figures 8(a) and 8(b)). However, the serum SCF level assay showed that no significant difference existed between the NC and MC groups (*P* > 0.05). The MD group presented relatively lower serum SCF concentrations than the other groups, although the differences were not significant (*P* > 0.05) (Figure 8(c)).

#### 3.1.7. Impact on c-Kit

**Figure 9:**
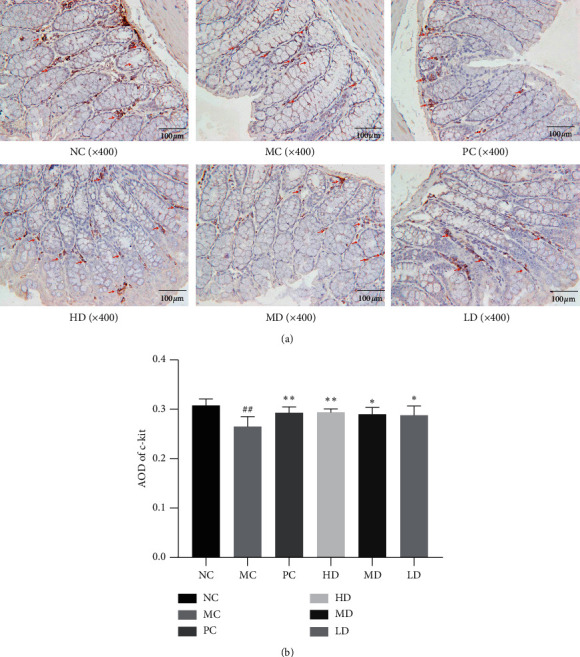
(a) Immunohistochemical analysis of c-kit. (b) AOD value of c-kit.NC group was considerably superior to MC group (0.308 ± 0.013 versus 0.265 ± 0.020 and ^##^*P* < 0.01). Similar to the result of SCF and PC, combined with HD group, have a significantly higher value compared with MC group (0.293 ± 0.012 versus 0.265 ± 0.020; ^*∗∗*^(*P* < 0.01); 0.294 ± 0.007 versus 0.265 ± 0.020 *P* < 0.01). MD and LD groups showed higher AOD value as well (0.290 ± 0.014 versus 0.265 ± 0.020; ^*∗*^*P* < 0.05; 0.288 ± 0.019 versus 0.265 ± 0.020 (*P* < 0.05).

Western blot analysis demonstrated that c-kit protein expression in the colon was significantly higher in the NC group than in the MC group (*P* < 0.01). The protein levels were higher in the PC and HD groups than in the MC group (*P* < 0.01), with no significant difference from the LD or MD group (*P* > 0.05) (Figures 7(a) and 7(c)). Immunohistochemical analysis results showed that c-kit-positive cells were scattered in the mesenchyme of colon glands. The MC group presented markedly reduced positive cells compared with the NC group. In addition, the PC and HD groups showed an apparent advantage in quantity. The MD group displayed more c-kit-positive cells than the LD group (Figures 9(a) and 9(b)).

#### 3.1.8. Aquaporin 4 Variation

Western blot analysis showed that the MC group expressed markedly higher levels of AQP4 than the NC group (*P* < 0.01). MD group protein concentrations were elevated (*P* < 0.05); meanwhile, PC and HD group protein concentrations showed the same effect (*P* < 0.01). However, no effect was found in the LD group (*P* > 0.05) (Figure 7(d)).

### 3.2. Discussion

STC refers to a condition featured by delayed emptying of the ascending and transverse colon with prolongation of transit [18]. Therefore, 24 h stool weight and ITR could be representative diagnostic indicators for this research. In this study, all mice had much lower fecal dry weight and ITR than those in the NC group after continuous injection of morphine, demonstrating that the STC model was successfully developed. In addition, high- and medium-dose GRG gavage mice had higher stool dry weight and ITR, suggesting that specific concentrations of GRG had a positive effect on relieving the symptoms of STC mice.

Receptors of motilin are distributed in muscle and myenteric plexus of the colon [19], and Ulusoy et al. found that children with functional constipation have lower serum motilin levels [7]. Mulberry apparently relieves the symptoms of diphenoxylate-induced constipation in mice and simultaneously increases the serum level of motilin [20]. However, few direct mechanistic research studies comparing STC and motilin have been conducted. We found that a high concentration of GRG was closely related with the elevated serum motilin concentration of STC mice. Medium and low concentrations of GRG showed little correlation with motilin, which indicated that the correlation between GRG and serum motilin may be dose-dependent. According to our ELISA results, mice that were administered morphine manifested significantly lower levels of motilin, which indicates that STC conditions may lead to low serum motilin levels in mice; nevertheless, the concrete relationship and mechanism between them still needs further research.

No consensus on the mechanism of STC has been achieved thus far. However, the quantity and density of ICCs in the colon have been widely reported to play an important role in the onset of STC [11, 12]. ICCs could be closely related to gastrointestinal motility and generate electrical activity, which could provide the primary drive for muscular contraction of the colon [21]. The c-kit/SCF signaling pathway is considered to be a classical pathway that could determine the quantity and viability of ICC. As important transmembranous receptors of ICC, c-kit protein is coded by protooncogene c-kit. Receptor tyrosine kinases (RTKs), the domain receptors of c-kit, are necessary for signal transmission between the ICC cytomembrane and the inside of the cell [22]. A previous study showed that an inhibitory c-kit antibody could reduce the ICC of mice and intestinal dynamics [23]. SCF, the ligand of the c-kit receptor, is a 30 kDa glycoprotein. There are two kinds of SCFs: membrane-bound isoforms and soluble isoforms. Both proteins can bind to c-kit, thereby playing important roles [24]. The connection between c-kit and SCF could activate the function of RTK. Therefore, inhibition of the c-kit/SCF pathway could influence the proliferation, differentiation, and growth of ICCs [25]. The results of our study showed that the colon tissue SCF and c-kit protein expression levels were lower in the MC group. c-kit-positive cells, combined with SCF-positive cells, were also reduced, indicating that 45 days of morphine injection altered c-kit and SCF expression and distribution in the mouse colon. The colon tissue SCF and c-kit protein expression increased in all the GRG groups after 14 days of treatment. No significant difference in therapeutic effect was found between high-dose GRG and mosapride. Immunohistochemical analysis showed that the quantity of SCF- and c-kit-positive cells was elevated by high- and medium-dosage GRG. Hence, we speculate that morphine's negative effect on the c-kit/SCF signal pathway of mice contributes to the reduction in ICC number, which further causes weakened gastrointestinal mobility and STC attack. GRG's curative effect on the symptoms of STC and ITR in the mouse model may be closely related to its restorative function on the c-kit/SCF pathway, which subsequently improves the quantity and function of ICC. In addition, SCF- and c-kit-positive cells were found mainly scattered within the mesenchyme of colon glands but little in the muscularis externa according to our result, the reason of which might need further research in the future.

Several studies [26–28] have reported that changes in serum SCF are closely associated with a series of respiratory, circulatory, and immunological diseases. The objective of the serum SCF assay was to explore the relationship between serum SCF levels and STC and the influence of GRG on this relationship. However, we found no significant difference among the six groups and thereby infer that there is no specific association between STC and serum SCF levels. We also speculate that the effect of GRG has no connection with mouse serum SCF levels.

Large amounts of aquaporins exist in the intestinal canal and in other organs. Aquaporins have been found to play an important role in water transport, through which water passes selectively and fluid homeostasis can be regulated [29]. Hence, changes in the level of aquaporins could change fecal water content and contribute to diarrhea or constipation. To date, a number of experiments have verified the relationship between AQP3 and constipation, drawing the conclusion that rats with STC, particularly morphine-induced constipation, express higher levels of AQP3 in the colon and subsequently promote water transport from the intestinal tract to the vasculature, which dries and hardens feces [30, 31]. However, little research has been conducted on other members of aquaporins. To further confirm the connection between aquaporins and STC and the specific mechanism of colonic water transport, other AQPs should be studied, and multiple animal models should be built. In contrast to other aquaporins, AQP4 is expressed mainly on mouse colonic mucosal epithelial cells [31]. A previous study [32] showed that mice with knockout of the AQP4 gene displayed higher water content in the feces, certifying that AQP4 on the colonic epithelium promoted transepithelial osmotic water permeability. Wang et al. [33] analyzed colonic mucosa protein in 45 STC patients by immunohistochemistry and found that AQP4 overexpression facilitates water transport and might be a significant factor in the pathogenesis of STC. In this study, the western blot results revealed that morphine injection for 45 days elevated the expression of AQP4 in colon tissue, which is in line with a previous opinion [29]. High-dose GRG and mosapride both showed obvious inhibitory effects on AQP4 expression, which may be one functional mechanism of the favorable therapeutic effects of the two drugs. No significant difference was found between low-dose GRG gavage mice and MC mice, indicating that the influence of GRG on AQP4 may be highly concentration dependent.

## 4. Conclusions

GRG improved the symptoms of morphine-induced STC mice, the potential mechanism of which may be associated with repairing the SCF/c-kit pathway and reducing the expression of AQP4 in the colon. GRG enhanced the serum motilin level of mice.

A connection between morphine-induced STC and the level of mouse serum SCF was not found, and GRG could not change STC mouse serum SCF levels.

## Data Availability

All data included in this study are available upon request to the corresponding author.
